# Metaheuristic-Driven Feature Selection for Human Activity Recognition on KU-HAR Dataset Using XGBoost Classifier

**DOI:** 10.3390/s25175303

**Published:** 2025-08-26

**Authors:** Proshenjit Sarker, Jun-Jiat Tiang, Abdullah-Al Nahid

**Affiliations:** 1 Electronics and Communication Engineering Discipline, Khulna University, Khulna 9208, Bangladesh; prosensarker4@gmail.com; 2 Centre for Wireless Technology, CoE for Intelligent Network, Faculty of Artificial Intelligence & Engineering, Multimedia University, Persiaran Multimedia, Cyberjaya 63100, Selangor, Malaysia

**Keywords:** human activity recognition, extreme gradient boosting, golden jackal optimization, war strategy optimization, feature extraction, SHAP, misclassifications

## Abstract

Human activity recognition (HAR) is an automated technique for identifying human activities using images and sensor data. Although numerous studies exist, most of the models proposed are highly complex and rely on deep learning. This research utilized two novel frameworks based on the Extreme Gradient Boosting (XGB) classifier, also known as the XGBoost classifier, enhanced with metaheuristic algorithms: Golden Jackal Optimization (GJO) and War Strategy Optimization (WARSO). This study utilized the KU-HAR dataset, which was collected from smartphone accelerometer and gyroscope sensors. We extracted 48 mathematical features to convey the HAR information. GJO-XGB achieved a mean accuracy in 10-fold cross-validation of 93.55% using only 23 out of 48 features. However, WARSO-XGB outperformed GJO-XGB and other traditional classifiers, achieving a mean accuracy, F-score, precision, and recall of 94.04%, 92.88%, 93.47%, and 92.40%, respectively. GJO-XGB has shown lower standard deviations on the test set (accuracy: 0.200; F-score: 0.285; precision: 0.388; recall: 0.336) compared to WARSO-XGB, indicating a more stable performance. WARSO-XGB exhibited lower time complexity, with average training and testing times of 30.84 s and 0.51 s, compared to 39.40 s and 0.81 s for GJO-XGB. After performing 10-fold cross-validation using various external random seeds, GJO-XGB and WARSO-XGB achieved accuracies of 93.80% and 94.19%, respectively, with a random seed = 20. SHAP identified that range_gyro_x, max_acc_z, mean_gyro_x, and some other features are the most informative features for HAR. The SHAP analysis also involved a discussion of the individual predictions, including the misclassifications.

## 1. Introduction

HAR is the automated process of identifying and classifying human actions from sensor data [1]. There is a considerable amount of research on the topic, which is mainly divided into two types: vision-based HAR and sensor-based HAR [2]. Vision-based HAR leverages machine vision techniques using image and video data to identify and classify human actions. In contrast, sensor-based HAR relies on signals captured from various sensors, such as accelerometers, gyroscopes, and magnetometers, to detect movement patterns and activities. In both approaches, the process typically begins with a proper data acquisition setup, followed by data preprocessing (including filtering), feature engineering, and finally classification for activity identification. Data collected from sensors is often affected by various types of noise, which can significantly degrade the quality and reliability of the signals [3]. Common sources of noise include environmental interference, sensor drift, motion artifacts, time delay, and electronic noise. Therefore, noise reduction is an essential step in processing sensor-based HAR datasets to enhance the accuracy of activity recognition. Recent developments in smartphones have made them ideal devices for data acquisition using their accelerometer and gyroscope sensors, as seen in the UCI HAR dataset [4], WISDM HAR [5], and RealWorld HAR [6], among others, as well as “KU-HAR: An Open Dataset for Human Activity Recognition”, a similar type of dataset [7]. In such datasets, data is typically collected within defined time windows and labeled with corresponding Person ID and Activity ID. For example, data collected from Person 01 performing Activity 01 may be labeled as PID_01_A01, combining both identifiers for effective organization and tracking.

HAR has numerous applications across diverse domains, including healthcare and assisted living, rehabilitation, sports and fitness tracking, workplace safety and ergonomics, smart home automation, surveillance and security, human–computer interaction, military and defense, and robotics [8]. Considering the scope of HAR, recent studies have focused on machine learning (ML) implementations of HAR using a variety of algorithms and datasets. Simpler models tend to yield lower accuracy, while larger and more complex neural networks have demonstrated a significantly higher performance, as shown in Table 1. For example, Sikder et al. utilized a traditional Random Forest (RF) model, achieving an accuracy of 89.67%, which highlights the potential of ensemble-based methods to capture complex activity patterns from sensor data [9]. Al-Qaness et al. combined an Aquila Optimizer (AO) with RF, achieving an accuracy of 88.53%, reflecting ongoing efforts to enhance the conventional models with modern metaheuristic optimizers [10]. More sophisticated deep learning approaches have improved the classification capabilities further. Akter et al. proposed an attention-mechanism-based deep learning feature combination (AM-DLFC) framework, achieving 96.86% accuracy, illustrating the effectiveness of attention mechanisms in focusing on the most informative segments of the input data [1]. Guo et al. developed a Transformer-based model with Relative Position Embedding (vRPE) and a Convolutional Feature Extractor Block (CFEB), yielding an accuracy of 96.80%, underscoring the Transformer architecture’s ability to model long-range dependencies in sequential data [11]. Pavliuk et al. introduced a hybrid architecture combining DenseNet121 with Morlet mother wavelet transformations (MWTs), achieving 97.48% accuracy. This combination of deep convolutional networks with a time–frequency analysis has proven highly effective in extracting both spatial and temporal features [2]. The highest reported accuracy comes from Kumar et al., who developed a Deep-HAR model, reaching an impressive 99.98% accuracy [12].

Complex deep learning models have often required substantial computational resources, extended training times, and high-performance hardware, leading to increased costs and delays. Moreover, the existing models have lacked explainable artificial intelligence, keeping their decision processes opaque. They have not revealed the most influential features, nor have they addressed or analyzed misclassifications. Additionally, traditional classifier-based models in previous studies have not achieved a notable performance. To address these challenges, we have proposed a more efficient and accessible approach to the HAR process by employing metaheuristic algorithms (MHAs) for feature optimization, combined with a traditional machine learning classifier, XGB, using the KU-HAR dataset. This approach effectively reduces the computational complexity while maintaining competitive classification performance. The key contributions of this study are as follows:

- This scientific research has introduced a simple and effective feature engineering strategy for preprocessing the KU-HAR dataset;
- This research has employed two different MHAs—Golden Jackal Optimization and War Strategy Optimization—for feature selection and dimensionality reduction;
- This work has demonstrated the effectiveness of a traditional classifier, XGB, in achieving a superior HAR performance in terms of accuracy, F-score, precision, recall, and Area Under the Curve (AUC);
- We have applied Shapley Additive Explanations (SHAP), an explainable AI technique, to interpret the model predictions and assess the feature importance, including an in-depth analysis of misclassifications.

So, to overcome the research gaps of previous studies, our proposed solutions can be summarized as (i) introducing proper and informative feature engineering; (ii) ensuring proper optimization of the hyperparameters of the classifier for a better performance; (iii) reducing the number of features to minimize the cost and enable fast decision-making; and (iv) elucidating the nature of the classifier through SHAP, including monitoring the feature importance.

The remainder of this research work has been organized into four major sections. Section 2: Materials and Methodsprovides a brief description of the dataset and the algorithms employed. The next section, Section 3, Results, presents the findings obtained from the proposed models. In Section 4, Discussion, we compare our models with the existing approaches and discuss the future scope of this research, including its limitations. Finally, Section 5, Conclusions, summarizes this study by highlighting its key findings.

## 2. Materials and Methods

**Figure 1:**
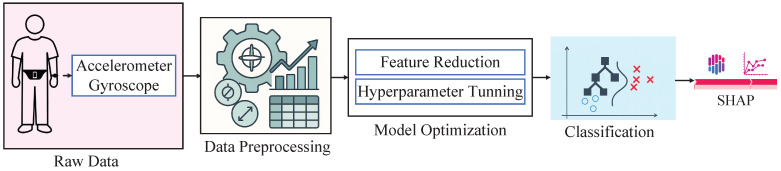
The research methodology of this research work.

In this research work, we have utilized the KU-HAR dataset. Next, we have applied a feature extraction technique based on mathematical expressions as part of the data preprocessing stage. Subsequently, we have deployed two proposed models: XGB optimized with the GJO algorithm (GJO-XGB) and XGB optimized with the WARSO algorithm (WARSO-XGB). Finally, we have performed an in-depth interpretability analysis of the models using SHAP, as illustrated in Figure 1.

### 2.1. Dataset

“KU-HAR: An Open Dataset for Human Activity Recognition” is a publicly available dataset in the Mendeley Data Repository, accessed on 26 June 2025. It was specifically developed to support the training and evaluation of machine learning models in recognizing human actions. The KU-HAR dataset contains sensor data from 90 participants (75 males and 15 females) from Khulna University, Khulna, Bangladesh, along with 18 distinct activities ranging from static postures (e.g., standing, sitting, lying) to dynamic movements (e.g., walking, running, jumping, stair navigation, and playing table tennis). The data have been collected using smartphone-based accelerometer and gyroscope sensors. The phone models used in the KU-HAR data acquisition process are Samsung Galaxy J7 (2017), Xiaomi Redmi Note 4, Realme 3 Pro, Realme 5i, and Realme C3. The accelerometer records the acceleration along the X, Y, and Z axes in meters per second squared (m/s^2^). On the other side, the gyroscope captures the rate of rotation around the X, Y, and Z axes in radians per second (rad/s). The raw output has been interpolated to maintain a constant sampling rate of 100 Hz, ensuring uniform time intervals between readings. The dataset includes 1945 raw activity samples, from which 20,750 subsamples have been extracted to support model training and evaluation. Each activity has been performed under standardized conditions—for example, walking 20 m, jumping 10 times, or sitting still for one minute—ensuring consistency and quality in the recorded data.

So, the original dataset comprises 20,750 rows and 1803 columns. For each row (or subsample), columns 1–900 contain accelerometer readings across the X, Y, and Z axes, while columns 901–1800 include gyroscope readings for the same axes. Thus, the features are located in columns 1 to 1800. Column 1801 specifies the activity class ID (ranging from 0 to 17) and serves as the target label. Columns 1802 and 1803 provide metadata, indicating the length of each channel’s data and the serial number of the subsample, respectively. Table 2 presents the data structure of the original dataset. And Table 3 displays the activities in the KU-HAR dataset and short descriptions.

### 2.2. Data Preprocessing

In the data preprocessing stage, we have applied a set of simple yet effective mathematical equations to extract meaningful features—a process commonly referred to as feature engineering [13]. In our study, we have adopted a simpler and more computationally efficient technique for feature extraction, rather than directly using the raw sensor signals from the original KU-HAR dataset. Specifically, we have transformed each time-series segment into a set of meaningful statistical features that effectively summarize the underlying patterns in the data. The extracted features for each signal axis include the mean, median, root mean square (RMS), minimum, maximum, standard deviation (SD), range, and mean absolute deviation (MAD). This feature engineering approach reduces the data dimensionality and helps the model to generalize better while significantly lowering the computational cost compared to that for models that rely on raw time-series data. These features were computed using the following formulas in Table 4.

After computing six statistical features for each sensor channel—namely the X, Y, and Z axes of both the accelerometer and the gyroscope—we obtained a total of 48 features per sample. By appending the corresponding target activity class to each sample, the final dataset consists of 49 columns and 20,750 rows. The following analysis was conducted on the newly transformed dataset. Table 5 shows the list of new features extracted through data preprocessing. After completing the feature extraction, we prepared a custom 10-fold validation dataset, maintaining 70% for training and 30% for testing at each fold.

### 2.3. The Extreme Gradient Boosting Classifier

The XGB classifier has been adopted to classify the target variable effectively. The prediction for a given input instance $x_{i}$ is formulated in Equation (1), where $Y_{i}$ denotes the model’s predicted output [14].

(1) $$Y_{i}=\phi \left(x_{i}\right)=\sum_{k=1}^{K}I_{k}\left(x_{i}\right),\;I_{k}\in F$$

In this formulation, each $I_{k}$ corresponds to an individual decision tree, and *F* denotes the space of all possible regression trees. The input vector $x_{i}$ is assumed to comprise independent features, and *K* represents the total number of additive trees in the ensemble. The overall objective function, including both the prediction error and regularization, is defined in Equation (2) [14].

(2) $$L_{0}\left(\phi \right)=\sum_{i}l\left(Y_{i},y_{i}\right)+\sum_{k}Reg\left(f_{k}\right)$$

Here, the loss term $l(Y_{i},y_{i})$ measures the discrepancy between the predicted output $Y_{i}$ and the true label $y_{i}$, while the regularization term $Reg(f_{k})$ controls the model complexity by penalizing the number of leaves and the magnitude of their weights.

### 2.4. The Metaheuristic Algorithm

MHAs have been widely used for both feature selection and hyperparameter tuning. Feature selection is the mathematical process of selecting the most influential subset of features by eliminating the non-influential features [15]. Recent researchers have shown the greater importance of developing newer optimizers daily [16,17,18]. In this research study, we used two different MHAs: GJO and WARSO.

#### 2.4.1. Golden Jackal Optimization

The GJO algorithm emulates the collaborative hunting behavior of golden jackals, particularly when hunting in pairs. Introduced by Chopra et al. in 2022 [19], this strategy capitalizes on the increased hunting efficiency of jackals when they operate in male–female pairs. The pair-hunting process consists of three main phases: (i) locating and approaching the prey, (ii) surrounding the prey, and (iii) launching an attack. The initialization of candidate solutions within the search space is expressed using Equation (3) [19].

(3) $$Y_{0}=Y_{\min}+\mathrm{rand}\times \left(Y_{\max}-Y_{\min}\right)$$

Here, $Y_{0}$ is the initial position of a candidate solution, while $Y_{\min}$ and $Y_{\max}$ represent the lower and upper bounds of the search variables, respectively. The term rand is a vector of uniformly distributed random numbers in the range [0,1]. During the exploration stage, the movement of the male and female jackals toward the prey is governed by Equations (4) and (5) [19].

(4) $$Y_{1}\left(t\right)=Y_{M}\left(t\right)-E\cdot \left|Y_{M}\left(t\right)-R_{levy}\cdot \mathrm{Prey}\left(t\right)\right|$$

(5) $$Y_{2}\left(t\right)=Y_{FM}\left(t\right)-E\cdot \left|Y_{FM}\left(t\right)-R_{levy}\cdot \mathrm{Prey}\left(t\right)\right|$$

In these expressions, $Y_{1}\left(t\right)$ and $Y_{2}\left(t\right)$ refer to the updated locations of the male and female jackals at iteration *t*. $Y_{M}\left(t\right)$ and $Y_{FM}\left(t\right)$ are their respective current positions, and Prey(t) indicates the prey’s location in the search space. The variable E denotes the energy level of the prey, which diminishes over time, while $R_{levy}$ is a random vector generated using the Levy flight mechanism. The candidate solution for the subsequent iteration is computed as the average of the male and female positions, as shown in Equation (6) [19].

(6) $$Y\left(t+1\right)=\frac{Y_{1}\left(t\right)+Y_{2}\left(t\right)}{2}$$

As the prey becomes more fatigued and its energy *E* drops, the jackals shift into the exploitation phase, wherein they encircle and leap toward the prey. This behavior is modeled using Equations (7) and (8) [19].

(7) $$Y_{1}\left(t\right)=Y_{M}\left(t\right)-E\cdot \left|R_{levy}\cdot Y_{M}\left(t\right)-\mathrm{Prey}\left(t\right)\right|$$

(8) $$Y_{2}\left(t\right)=Y_{FM}\left(t\right)-E\cdot \left|R_{levy}\cdot Y_{FM}\left(t\right)-\mathrm{Prey}\left(t\right)\right|$$

Even in the exploitation stage, the final updated solution is obtained using Equation (6). The transition between exploration and exploitation is decided by the magnitude of *E*: if |E|≥1, the algorithm remains in the exploration phase; otherwise, it enters the exploitation phase.

#### 2.4.2. War Strategy Optimization

WARSO is one type of MHA that follows the ancient war strategy proposed by Ayyarao et al. in 2022 [20]. Ancient war has several tactical steps, such as Random Attack, Attack Strategy, Signaling by Drum, Defense Strategy, the replacement of weak soldiers, and Traps by the Opposition. The attack strategy position update for a soldier *i* is mathematically modeled as Equation (9) [20].

(9) $$P_{i}\left(t+1\right)=P_{i}\left(t\right)+2\times \rho \times \left(C-King\right)+R\times \left(W_{i}\times King-P_{i}\left(t\right)\right)$$

where $P_{i}\left(t\right)$ represents the current position of the soldier, *C* denotes the commander’s position, King is the king’s position, ρ controls the exploration–exploitation balance, R is a uniform random number in [0,1], and $W_{i}$ is the soldier’s adaptive weight. The term 2ρ(C−King) drives coordinated movement toward strategic positions, while $R(W_{i}King-P_{i}\left(t\right))$ introduces stochastic exploration scaled by the soldier’s weight. This formulation dynamically balances offensive maneuvers with positional adaptability during battlefield advancement. The position update acceptance criterion is defined by Equation (10) [20].

(10) $$P_{i}\left(t+1\right)=\left[P_{i}\left(t+1\right)\right]\times \left(F_{n}\geq F_{p}\right)+\left[P_{i}\left(t\right)\right]\times \left(F_{n}<F_{p}\right)$$

where $F_{n}$ is the fitness (attack force) at the new position, and $F_{p}$ is the fitness at the previous position. This conditional update ensures soldiers retain their positions only when the fitness improves or remains constant, preventing detrimental moves. Soldiers revert to their prior positions when fitness degrades, mimicking battlefield tactics where troops consolidate gains during unfavorable engagements. The rank $R_{i}$ of soldier *i* is updated according to Equation (11) [20].

(11) $$R_{i}=\left[R_{i}+1\right]\times \left(F_{n}\geq F_{p}\right)+\left[R_{i}\right]\times \left(F_{n}<F_{p}\right)$$

This promotion system increments the soldier’s rank $R_{i}$ by 1 upon successful fitness improvements ($F_{n}\geq F_{p}$), rewarding effective maneuvers. Unsuccessful moves leave the ranks unchanged, maintaining hierarchy stability. Rank accumulation signifies veteran status, influencing future weight adjustments and position updates. The soldiers’ weights are nonlinearly updated via Equation (12) [20].

(12) $$W_{i}=W_{i}\times {\left(1-\frac{R_{i}}{\mathrm{Max}\_\mathrm{iter}}\right)}^{\alpha }$$

where $R_{i}$ is the current rank, Max_iter is the maximum iterations, and α controls the decay rate. The weights decrease exponentially with rank progression and iteration advancement, initially promoting large exploratory steps ($W_{i}>1$) for battlefield reconnaissance and then transitioning to fine-grained exploitation ($W_{i}<1$) for precision targeting during the later stages of war. The exponent α modulates this exploration-to-exploitation shift. The defense strategy position update incorporates Equation (13) [20].

(13) $$P_{i}\left(t+1\right)=P_{i}\left(t\right)+2\times \rho \times \left(King-P_{R}\left(t\right)\right)+R\times W_{i}\times \left(c-P_{i}\left(t\right)\right)$$

Here, $P_{R}\left(t\right)$ denotes a randomly selected soldier’s position, enhancing the exploration diversity. The term $2\rho (King-P_{R})$ creates protective formations around the king, while $R\cdot W_{i}\left(c-P_{i}\right)$ maintains cohesion with the commander *c*. This formulation models defensive tactics where troops dynamically reconfigure to shield the leadership while scouting unknown territories. Weak soldiers are randomly relocated using Equation (14) [20].

(14) $$P_{w}\left(t+1\right)=Lb+R\times \left(Ub-Lb\right)$$

where Lb and Ub define the search space boundaries. This “replacement” strategy reinvigorates the army by repositioning under-performing soldiers ($P_{w}$) uniformly across the battlefield, preventing stagnation. Though disruptive, this approach provides fresh exploration vectors but risks destabilizing convergence. A superior relocation strategy guides weak soldiers, as shown by Equation (15).

(15) $$P_{w}\left(t+1\right)=-\left(1-R\right)\times \left(P_{w}\left(t\right)-\mathrm{median}\left(P\right)\right)+King$$

where R is a normally distributed random number, median(P) is the army’s median position, and King is the king’s position. This “retraining” approach pulls weak soldiers toward the collective center while retaining king alignment, preserving morale and knowledge. The Gaussian term R adds controlled randomness, yielding faster convergence than random replacement in empirical tests.

#### 2.4.3. Justification of the Choice of the Classifier and Optimizers

In this research work, we have focused on boosting-based classifiers. However, there are different types of boosting-based classifiers, such as Light Gradient Boosting Machine (LGBM), Categorical Boosting (CB), Gradient Boosting (GB), XGB, and some others. The XGB algorithm, developed by Tianqi Chen and Carlos Guestrin, is a highly scalable tree boosting system widely used by data scientists to achieve a state-of-the-art performance across ML tasks [14]. Previous studies have shown that XGB is a scalable ensemble method that has proven to be a dependable and effective solution for various ML tasks [21].

To choose the classifier, we applied multiple boosting-based classifiers to the preprocessed dataset without any optimization or cross-validation. Table 6 shows the prior evaluation performance of four different classifiers. Among them, XGB and LGBM showed the same highest training F-score (100%). However, XGB gained the highest test F-score of 92.55% among all of the models. Based on this prior evaluation, we selected XGB as the classifier for this research work.

Now, for the optimizer, we have selected GJO and WARSO. GJO and WARSO are comparatively new optimizers, proposed in 2022. GJO has been evaluated using 22 benchmark functions combining unimodal, multimodal, and fixed-dimension multimodal functions [19]. GJO has also been evaluated using engineering designs (e.g., welded beam design, tension compression spring design, and pressure vessel design) [19]. The evaluation results confirm the compatibility of GJO compared to that of other well-known optimizers. On the other hand, WARSO has been evaluated using a benchmark of 50 functions and 4 engineering problems [20]. The results have proven the potential of WARSO. As a result, we were motivated to select GJO and WARSO as the optimizers for this research work.

#### 2.4.4. Optimizer Problem Development

We have defined our custom problem combining the XGB classifier and the MHAs. During optimization, we used the F-score as a cost function, the fitness value of the agent participation process. First, we declared the boundary conditions for the hyperparameters and the feature range, as shown in Table 7.

The optimization process is executed independently for each fold in the training set. Each MHA is employed with a population size of 30 and 50 iterations (epochs). At each iteration, different candidate solutions—comprising combinations of selected features and hyperparameter configurations—are evaluated. The F1-score is used as the fitness function to measure the quality of each candidate. For a given iteration τ, the highest scoring candidate is denoted as $S_{\tau }$, while the overall best solution across all iterations is represented as $S^{*}$. This globally optimal solution encapsulates the optimal hyperparameter set ($H^{*}$) and the most informative feature subset ($F^{*}$). The full optimization routine is detailed in Algorithm 1.
**Algorithm 1** MHA-based optimization for each training fold1:**  Initialize optimization process**2:**  Step 1: Define search space constraints (B)**3:       Hyperparameter bounds for XGB:4:          $P_{1}$: Number of estimators: 100 to 3005:          $P_{2}$: Learning rate: 0.001 to 0.26:          $P_{3}$: Maximum tree depth: 3 to 77:          $P_{4}$: Minimum child weight: 1 to 108:          Number of selected features: 1 to 489:       Configure MHA with population = 30 and iterations = 5010:**Step 2: Iterative optimization**11:** for**τ=1 to 50 **do**12:        Generate feature subset $F_{\tau }$13:        Generate hyperparameter set $H_{\tau }=\left\{P_{1}^{\tau },P_{2}^{\tau },P_{3}^{\tau },P_{4}^{\tau }\right\}$14:        Train XGB using $F_{\tau }$ and $H_{\tau }$15:        Evaluate F1-score as fitness: $S_{\tau }$16:**end for**17:**Step 3: Global best identification**18:    Determine $S^{*}=\max\left\{S_{1},S_{2},\ldots ,S_{50}\right\}$19:    Extract $F^{*}$ and $H^{*}$ from $S^{*}$20:**End optimization**

Since the dataset has been divided into 10 folds for cross-validation, the optimization process has been independently performed 10 times, once for each fold. Each execution of the optimizer has produced a distinct combination of selected feature subsets and optimal hyperparameter settings based on the corresponding training data. As a result, a total of 10 optimized configurations have been obtained, consisting of 10 sets of feature selections and 10 associated hyperparameter combinations.

To finalize a single, unified configuration for model development, an aggregation strategy has been applied to consolidate the outcomes from all folds. For hyperparameter selection, a majority voting technique has been used. In this approach, for each hyperparameter (e.g., learning rate, maximum depth, number of estimators), the value that has occurred most frequently across the 10 folds has been selected as the final choice. If the majority identity is absent for any parameter, we have taken the value observed in the first fold. This has ensured that the selected hyperparameters represent the most consistently optimal values observed throughout the optimization runs. For feature selection, a union-based approach has been adopted. All features that have been selected in at least 1 of the 10 folds have been included in the final feature set. This strategy has allowed the model to incorporate all relevant features identified during the cross-validation process, thus enhancing its generalization capability.

#### 2.4.5. Performance Evaluation

The effectiveness of the models has been evaluated using five primary performance metrics: accuracy, F-score, precision, recall, and AUC. In addition, the computational cost has been analyzed in terms of two aspects: training time (TrT) and testing time (TsT) complexity. The mathematical expressions used to compute these evaluation metrics are provided below:

(16) $$\mathrm{Accuracy}=\frac{\mathrm{TP}+\mathrm{TN}}{\mathrm{TP}+\mathrm{TN}+\mathrm{FP}+\mathrm{FN}}$$

(17) $$\mathrm{Precision}=\frac{\mathrm{TP}}{\mathrm{TP}+\mathrm{FP}}$$

(18) $$\mathrm{Recall}=\frac{\mathrm{TP}}{\mathrm{TP}+\mathrm{FN}}$$

(19) $$F-\mathrm{score}=\frac{2\times \mathrm{Precision}\times \mathrm{Recall}}{\mathrm{Precision}+\mathrm{Recall}}$$

In these equations, true positive (TP) refers to the number of correctly predicted positive cases (e.g., heart attack patients), while true negative (TN) denotes correctly predicted negative cases. False positive (FP) represents negative samples incorrectly predicted as positive, and false negative (FN) corresponds to positive samples that have been incorrectly classified as negative.

#### 2.4.6. SHAP Explanation

SHAP is a model interpretability approach introduced by Lundberg et al. [22], which is grounded in the concept of Shapley values from cooperative game theory developed by Lloyd Shapley [23]. SHAP assigns a contribution value $\phi_{i}$ to each feature *i*, computed using the following formulation:

(20) $$\phi_{i}=\sum_{S\subseteq F\setminus \{i\}}\frac{|S|!(|F|-|S|-1)!}{|F|!}\left[f_{S\cup \{i\}}\left(x_{S\cup \{i\}}\right)-f_{S}\left(x_{S}\right)\right]$$

Here, *F* denotes the complete set of features, *S* is any subset of *F* that does not contain the feature *i*, and $f_{S}\left(x_{S}\right)$ indicates the model’s prediction using only the features in *S*. The term $f_{S\cup \{i\}}\left(x_{S\cup \{i\}}\right)$ represents the output when feature *i* is included in the subset.

The overall model prediction for an input instance *x* can be represented as the sum of the expected value of the model and the contribution of each feature:

(21) $$f\left(x\right)=E\left[f\left(x\right)\right]+\sum_{i=1}^{n}\phi_{i}$$

If the resulting value f(x) exceeds 0.5, the model predicts the instance as Class 1 (e.g., dengue-positive); otherwise, it is classified as Class 0.

## 3. Results

The result section has been organized into two main sub-sections: the optimization outcomes and the classification outcomes. Optimization outcomes describe the feature subsets and hyperparameter sets found in each fold optimization. However, the classification outcomes show the results regarding the final models and their activity recognition performance.

### 3.1. Optimization Outcomes

Since the dataset has been divided into 10 folds for cross-validation, the optimization process has been independently performed 10 times, once for each fold. Each execution of the optimizer has produced a distinct combination of selected feature subsets and optimal hyperparameter settings based on the corresponding training data. As a result, a total of 10 optimized configurations have been obtained, consisting of 10 sets of feature selections and 10 associated hyperparameter combinations, as presented in Table 8. This table shows the fold-wise feature subsets, along with the corresponding hyperparameters—the number of estimators ($P_{1}$), learning rate ($P_{2}$), maximum tree depth ($P_{3}$), and minimum child weight ($P_{4}$)—that have been identified throughout the optimization runs for both the GJO and WARSO frameworks.

The consolidated results of these final configurations have been summarized in Table 9. For the GJO optimizer, the finalized model includes 23 features, with the hyperparameters set at 280 estimators, a learning rate of approximately 0.116, a maximum depth of 7, and a minimum child weight of 1. Meanwhile, the final model for WARSO has incorporated a larger feature set of 44 features, alongside hyperparameters of 230 estimators, a learning rate of 0.2, a maximum depth of 6, and a minimum child weight of 2. These finalized parameters reflect the cumulative findings of the optimization procedure and provide the foundation for the final model training and evaluation.

### 3.2. Classification Outcomes

Classification using XGB and the finalized features and hyperparameters from GJO has been defined as GJO-XGB, while XGB with WARSO has been defined as WARSO-XGB. Table 10 presents the fold-wise performance across the 10 folds. Both models have demonstrated a perfect performance on the training data across all folds, consistently achieving 100% accuracy, F-scores, precision, and recall. This indicates that the models have fully fit the training sets, highlighting the effectiveness of the selected features and hyperparameters in modeling the training patterns. In the testing phase, WARSO-XGB exhibited a slightly better performance than that of GJO-XGB in most folds. The test accuracy for WARSO-XGB ranges from 93.65% to 94.47%, whereas GJO-XGB’s accuracy varies from 93.35% to 93.93%. Additionally, WARSO-XGB has consistently shown higher F-score, precision, and recall values across several folds, suggesting improved predictive reliability on unseen data.

**Figure 2:**
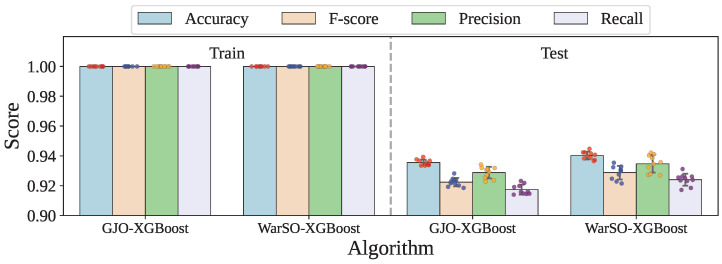
The performance distribution across the fold training and testing of the optimized models.

Figure 2 and Table 11 present the standard deviation values for the performance metrics obtained over 10-fold validation for both the GJO-XGB and WARSO-XGB models. As seen, both models have shown zero variation in their training performance across folds, confirming their perfect consistency on the training data. However, on the test data, WARSO-XGB exhibits slightly higher standard deviation across all metrics compared to that for GJO-XGB.

**Figure 3:**
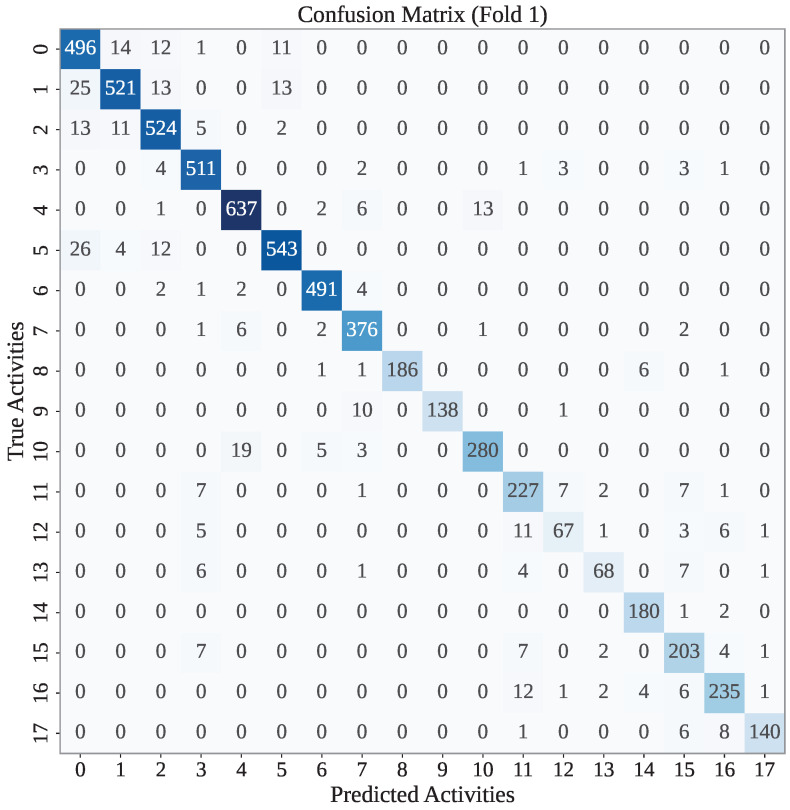
The confusion matrix of the first fold of the GJO-XGB model.

In each fold, our models have made some misclassifications. Here, we have presented the confusion matrix for the first fold of each framework. Figure 3 presents the confusion matrix found for the GJO-XGB framework in the first fold. According to Figure 3, GJO-XGB has mostly classified the activities correctly. For example, the test dataset for fold 1 has 507 activities notated as class 0, among which the model has correctly classified 496 instances. The remaining instances were misclassified into nearby classes such as 1, 2, and 5, indicating some feature-level similarities between these activities. Similarly, other classes like 2, 4, and 5 also show a strong performance, with 524, 637, and 543 correct predictions, respectively. So, among the 6225 records, GJO-XGB has correctly classified 5823.

**Figure 4:**
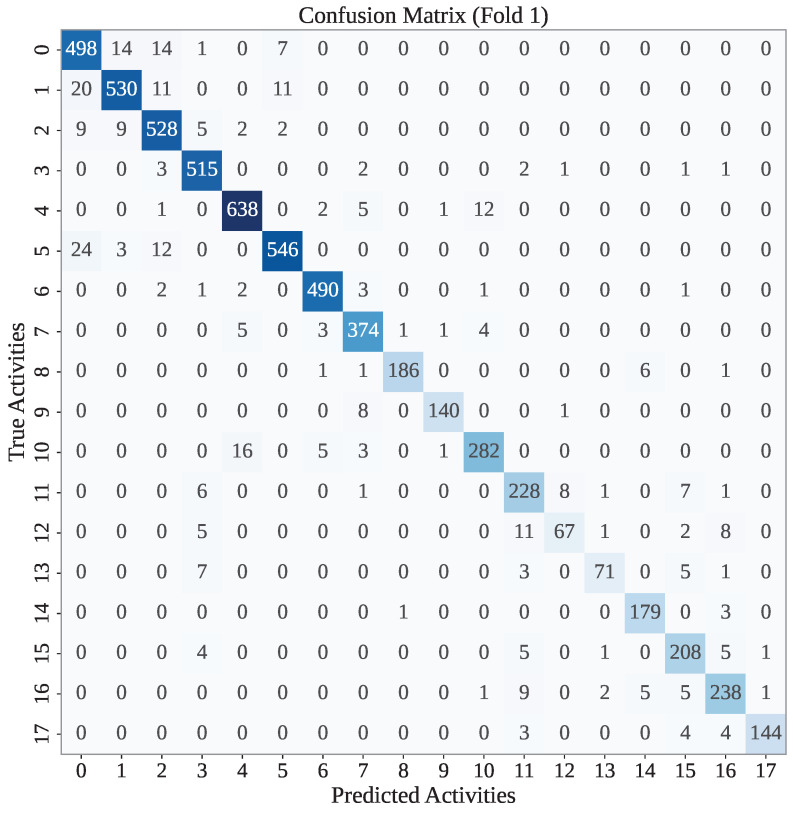
The confusion matrix of the first fold of the WARSO-XGB model.

Figure 4 presents the confusion matrix for the first fold of the WARSO-XGB framework. As shown in the figure, WARSO-XGB has achieved a high classification accuracy for most activity classes, with strong diagonal dominance in the matrix. For instance, out of 507 instances of activity 0, the model has correctly classified 498, while the rest were misclassified into similar classes, such as 1, 2, and 3. Similarly, classes 4, 5, and 2 have high correct classification counts of 638, 546, and 528, respectively, indicating that the model effectively distinguishes these activities. But WARSO has correctly classified 5860 records out of 6225 in the first fold test set.

**Figure 5:**
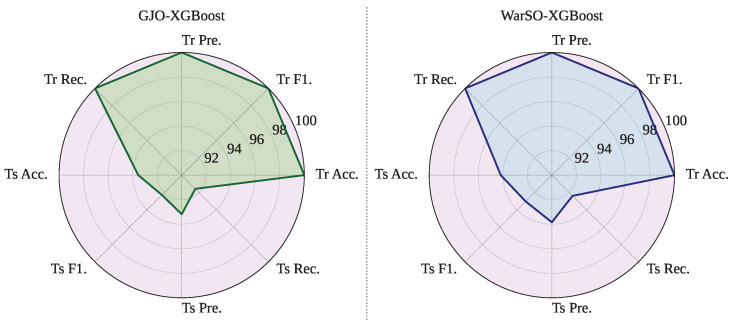
Mean performance of the proposed frameworks: GJO-XGB and WARSO-XGB.

However, WARSO-XGB has demonstrated the highest average test accuracy of 94.02%, outperforming GJO-XGB, which achieved 93.55%, as shown in Table 12. Additionally, WARSO-XGB achieved a higher mean F-score (92.88% vs. 92.24%) and test recall (92.40% vs. 91.74%), suggesting better consistency in identifying positive cases. In contrast, GJO-XGB has reported higher test precision (92.88%) compared to that for WARSO-XGB (93.47%), indicating a slightly better ability to reduce false positives. Figure 5 visualizes the side-by-side performance of the proposed two frameworks.

**Figure 6:**
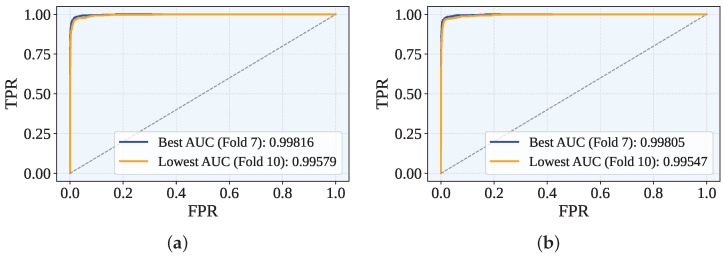
The best and worst ROC across the folds for both the GJO-XGB and WARSO-XGB models. (**a**) GJO-XGB ROC curves. (**b**) WARSO-XGB ROC curves.

Figure 6 displays the Receiver Operating Characteristic (ROC) curves for the best- and worst-performing folds, where the ROC curve visualizes the trade-off between the true positive rate (TPR) and the false positive rate (FPR) across classification thresholds. The AUC metric, which quantifies the overall discriminative ability (with 1.0 indicating perfect separation), reached peak values at fold 7 for both proposed frameworks (GJO-XGB: 0.99816; WARSO-XGB: 0.99805). The weakest performance occurred at fold 10 (GJO-XGB: 0.99579; WARSO-XGB: 0.99547). As summarized in Table 13, the mean AUC across all folds was marginally higher for GJO-XGB (0.99709) than WARSO-XGB (0.99699).

**Figure 7:**
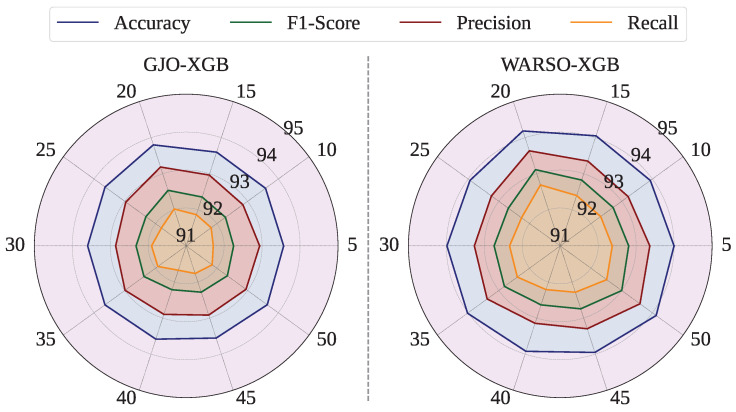
Mean test performance of GJO-XGB and WARSO-XGB for multiple random seeds.

To ensure the robustness and generalizability of our findings, we initially conducted the entire experimental process using a fixed random seed of 42 to generate consistent fold sets for cross-validation. In response to a reviewer’s suggestion, we extended our evaluation further by performing external iterations of 10-fold cross-validation using multiple random seeds: 5, 10, 15, 20, 25, 30, 35, 40, 45, and 50. These additional iterations introduced variability into the training and testing splits, thereby enabling a more comprehensive assessment of the model performance across different data partitioning scenarios. Figure 7 shows the 10-fold cross-validation mean test performance of each random seed. For GJO-XGB, the highest mean test accuracy among the random seeds was measured as 93.80%, which is higher than the accuracy reported in Table 12. Additionally, the mean test accuracies varied between 93.56% (random seed = 45) and 93.80% (random seed = 20) for GJO-XGB. However, the mean test performance for these new random seed settings have from 93.92% (random seed = 40) to 94.19% (random seed = 20). Table 14 shows the performance of different external random seeds for the fold sets.

### 3.3. Time Complexity

**Figure 8:**
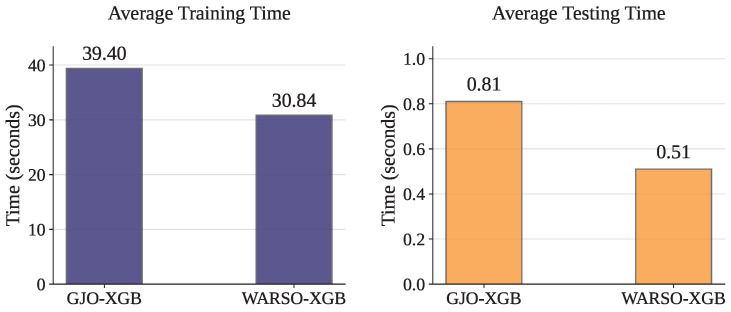
Computational time complexity showing the average training and testing times of the models.

Once the models have been optimized, they do not require any further optimization. Therefore, the time complexity was analyzed solely for the training and testing phases. Table 15 and Figure 8 present the training and testing times for each fold, along with the corresponding mean values. GJO-XGB recorded a mean training time of 39.40 s while handling 14,525 records and 23 features. In contrast, WARSO-XGB required only 30.84 s on average to train the models, despite using the same number of records with a larger feature set of 44. Moreover, WARSO-XGB consistently demonstrated lower testing times, achieving a mean of just 0.51 s, outperforming GJO-XGB in both aspects of execution time.

### 3.4. SHAP Analysis

**Figure 9:**
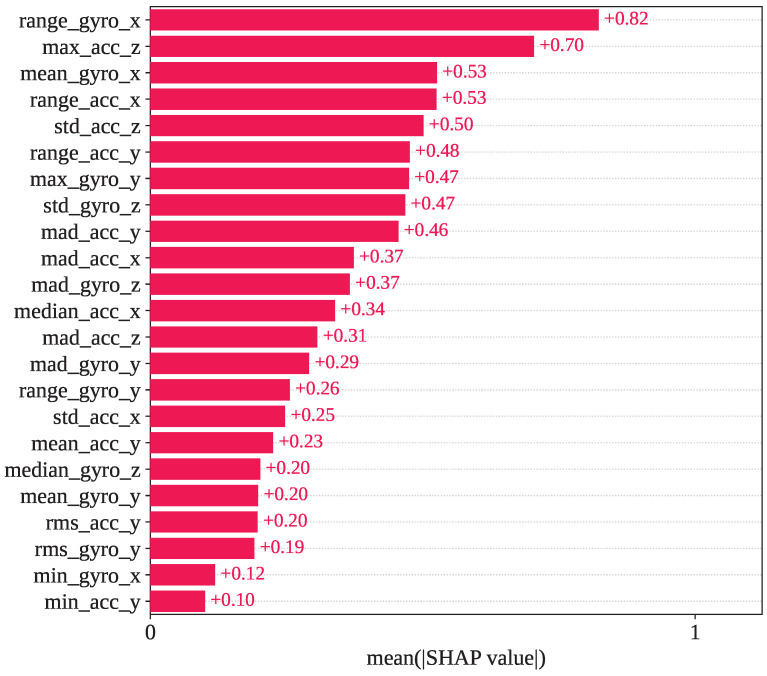
Mean SHAP values observed in the GJO-XGB model for the selected 23 features.

Figure 9 presents the mean SHAP value for the GJO-XGB framework. In test fold set 1, range_gyro_x has been recognized as the most impacted feature, with a mean SHAP value of +0.82. max_acc_z, mean_gyro_x, range_acc_x, and std_acc_z are the subsequent most influential features identified by SHAP, having mean SHAP values of +0.70, +0.53, +0.53, and +0.50, respectively.

**Figure 10:**
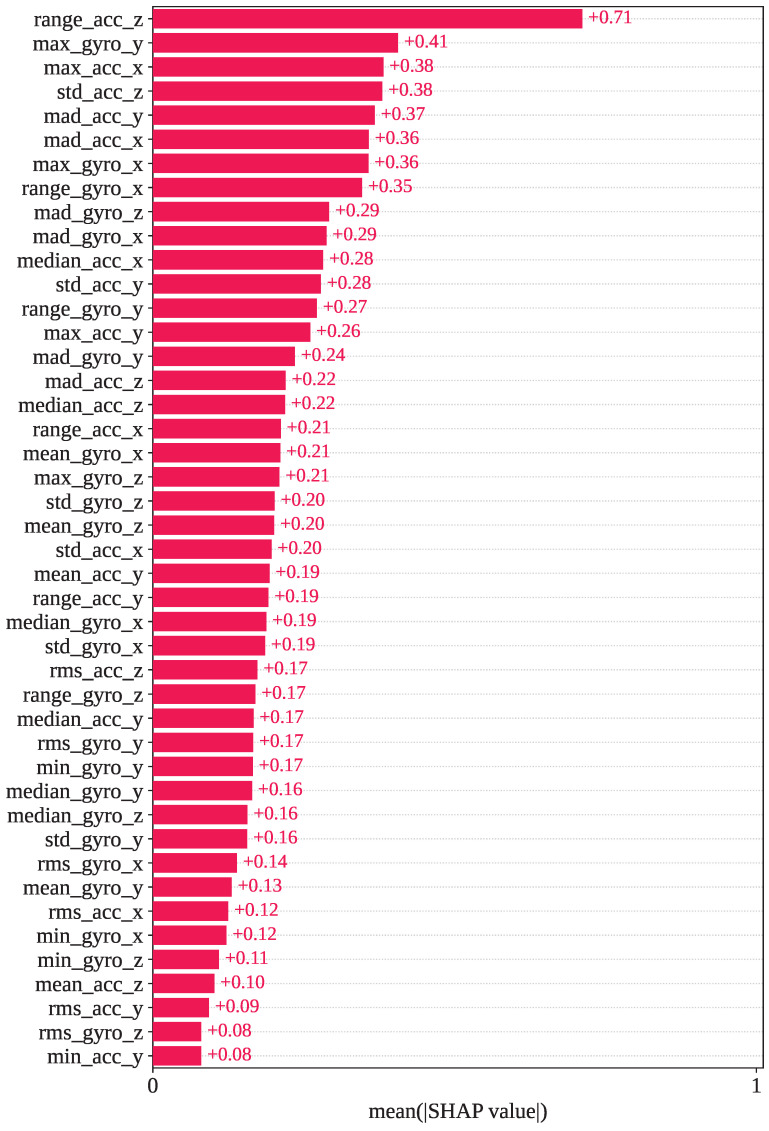
Mean SHAP values observed in the WARSO-XGB model for the selected 44 features.

However, Figure 10 shows the mean SHAP values calculated using SHAP for the WARSO-XGB framework. In WARSO-XGB, range_acc_z, max_gyro_y, max_acc_x, std_acc_z, and mad_acc_y are the top five most important features, which have mean SHAP values of +0.71, +0.41, +0.38, +0.38, and +0.37, respectively. We also analyzed individual predictions from the fold 1 test set, a correct prediction, and a wrong prediction, through the SHAP decision plot. The first sample in the fold 1 test set has an index of 17,578, and the first misclassified index is 5743. These two indices showed the same type of classification in both GJO-XGB and WARSO-XGB: index: 17,578, correct classification (actual activity: 6); index: 5743, wrong classification (actual activity: 12; predicted activity: 13).

**Figure 11:**
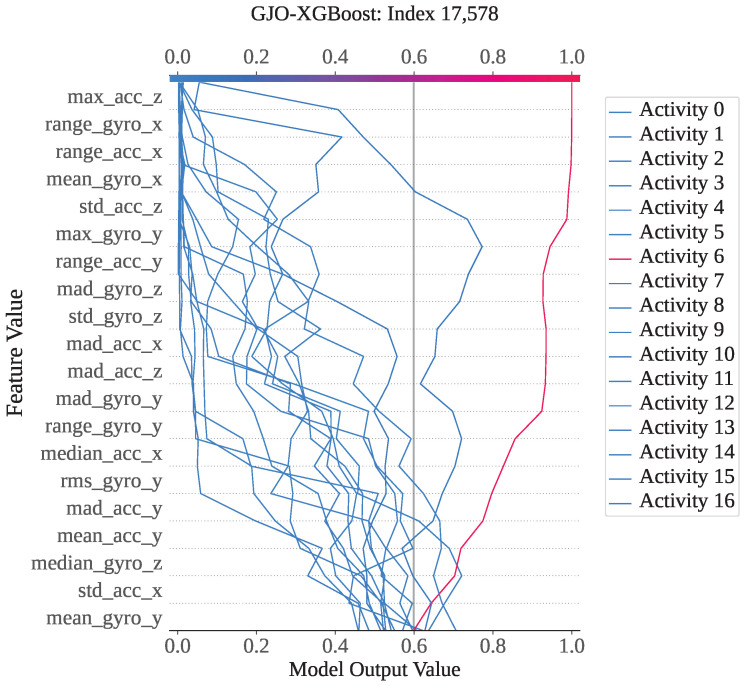
The GJO-XGB decision plot for a correct prediction (index: 17,578).

**Figure 12:**
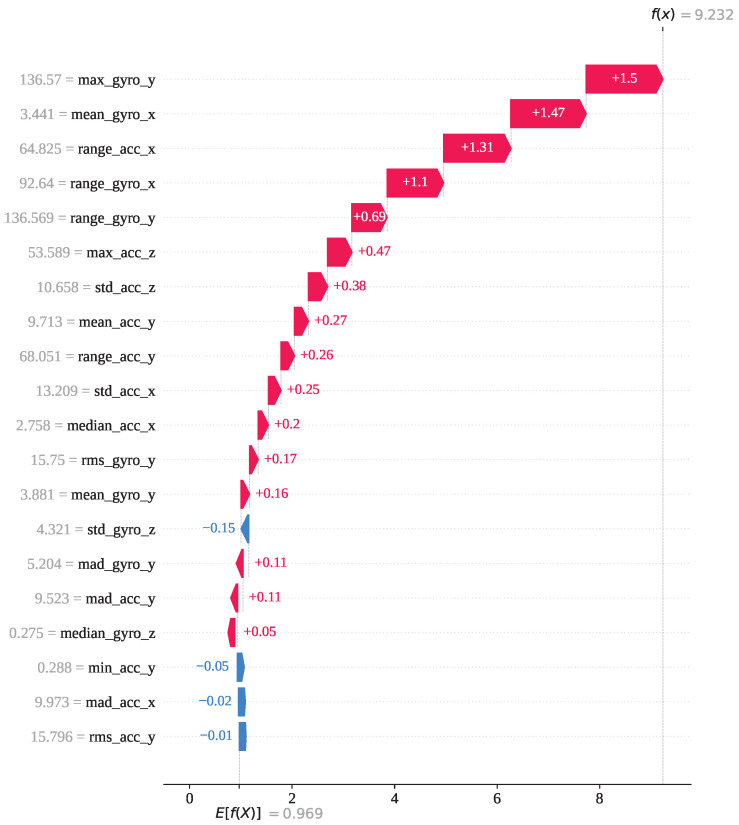
The GJO-XGB waterfall plot for a correct prediction (index: 17,578) for activity class 6.

For index 17,578, the sample belongs to activity class 6 (lay–stand), and the model correctly predicted it as class 6. As shown in Figure 11, activity 6 has the highest SHAP output value of approximately 1. The decision plot indicates that the SHAP values for all classes vary between 0 and 1, with activity 6 standing out prominently. This confirms that this instance is strongly associated with class 6, and therefore, GJO-XGB has accurately classified it. For a deeper analysis of this particular prediction, we have presented the waterfall plot for activity class 6. Figure 12 illustrates how each feature contributes to the final prediction, with the SHAP values indicating their influence in pushing the model output toward class 6. The feature max_gyro_y (136.57) has contributed the highest SHAP value of +1.5, followed by mean_gyro_x (+1.47), range_acc_x (+1.31), range_gyro_x (+1.10), and range_gyro_y (+0.69), among others. Collectively, these feature contributions have shifted the base value of the model output, E[f(x)]=+0.969, to a final SHAP output of f(x)=+9.232, which is higher than 0.5, thereby supporting correct classification of the instance as activity 6.

**Figure 13:**
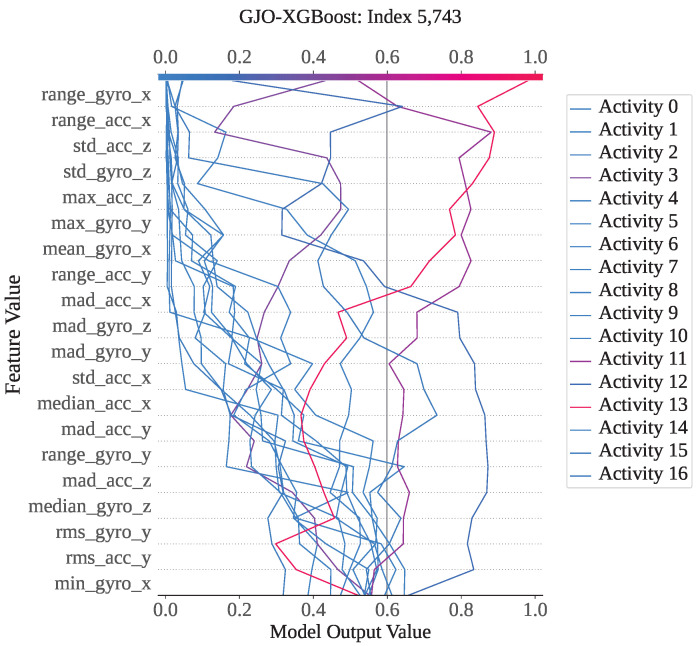
GJO-XGB decision plot for a wrong prediction (Index: 5743).

**Figure 14:**
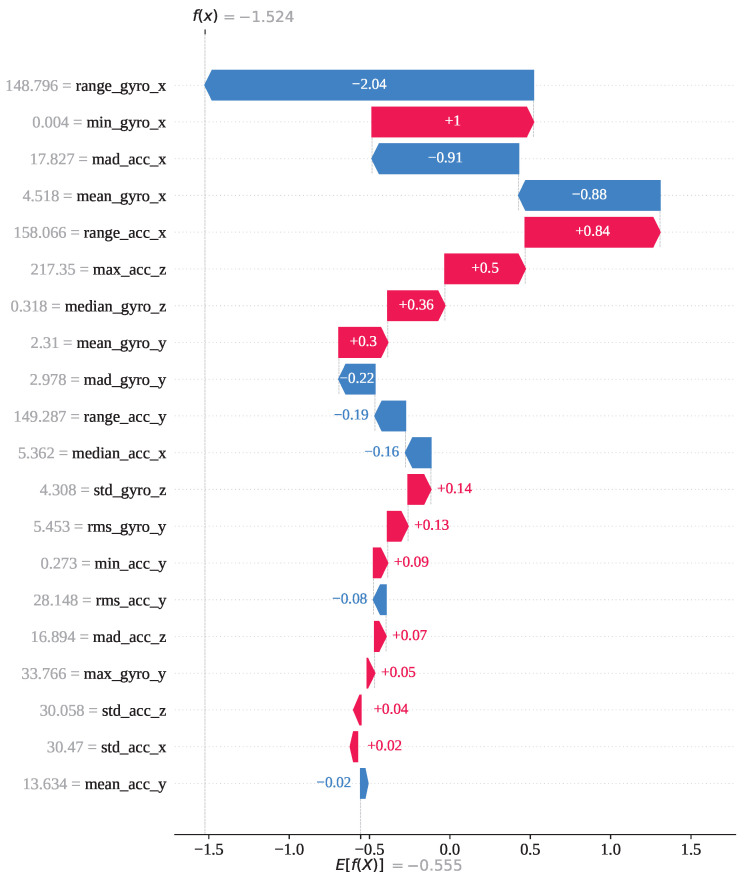
The GJO-XGB waterfall plot for a wrong prediction (index: 5743) for activity class 12.

Index 5743 is an instance from activity class 12; however, both GJO-XGB and WARSO-XGB classified it as activity 13, making the wrong prediction. Figure 13 shows that activity 13 has the highest model output value of 1 compared to the other activities. As a result, the classifier models have misclassified it as activity 13, though it is not. Figure 14 presents the waterfall plot for index 5743 from the perspective of activity 12 to understand why the model has not predicted it as activity 12. The feature range_gyro_x has been identified as the main effector for this misclassification, having a SHAP value of −2.04, and has also been influenced by mad_acc_x (−0.91) and mean_gyro_x (−0.88). Thus, the algebraic sum of the SHAP value f(x) has become −1.524, and the prediction has differed from activity 12.

**Figure 15:**
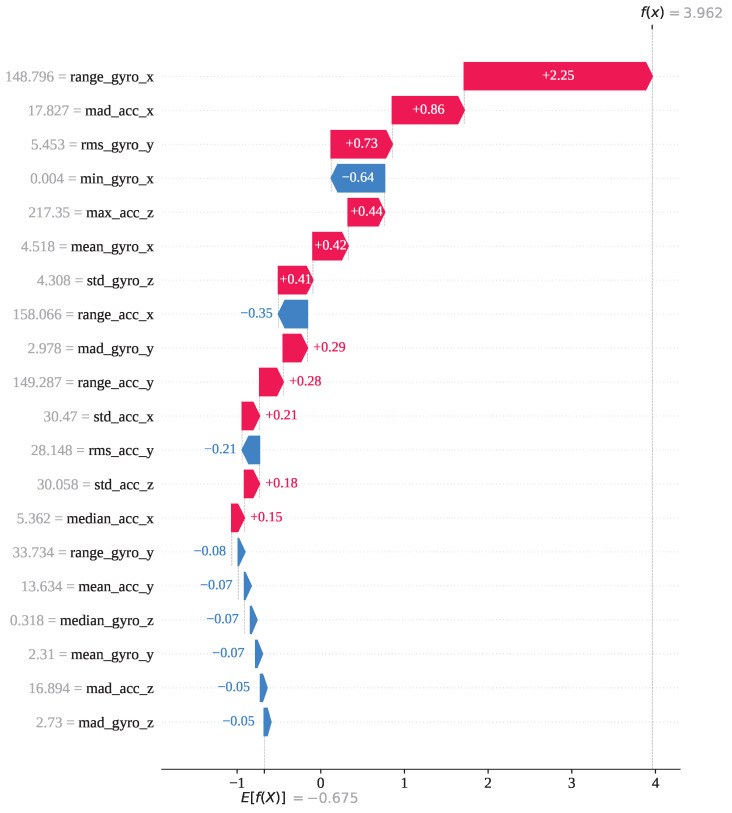
The GJO-XGB waterfall plot for a wrong prediction (index: 5743) for activity class 13.

Figure 15 presents the waterfall plot for the same index (5,743) from the perspective of activity 13 to understand the underlying reasons behind the model’s misclassification. The feature range_gyro_x has been identified as the most influential contributor, with a SHAP value of +2.25, followed by mad_acc_x (+0.86), mean_gyro_x (+0.73), max_acc_z (+0.44), and a few other features with positive SHAP values. Collectively, these contributions have shifted the base value E[f(x)]=−0.675 to a final SHAP output of f(x)=+3.96 for activity 13, while the output SHAP value for activity 12 is f(x)=−1.524. As a result, the model favored activity 13, leading to an incorrect classification. So, index 5743 has been mainly misclassified due to the impact of the features range_gyro_x, mad_acc_x, and mean_gyro_x.

## 4. Discussion

This section presents a comparative analysis between our proposed models and existing approaches, as well as a discussion on future scopes and potential limitations. Table 16 summarizes the performance of traditional classifiers, deep learning models, and our proposed frameworks on the KU-HAR dataset. The traditional classifier models [9,10] have shown a relatively moderate performance. The Random Forest [9] achieved 89.67% accuracy with an F-score of 87.59%, while the AO + RF model [10] obtained 88.53% accuracy. These results suggest that classical machine learning models, even when combined with feature selection techniques, struggled to exceed the 90% accuracy mark on this dataset. This limited performance is largely due to the inherent limitations of traditional classifiers. These models rely heavily on manually crafted statistical features, which may fail to capture the full complexity of human activity signals. Furthermore, they are prone to performance degradation due to inter-subject variability and class imbalance. Their static decision boundaries also limit their ability to generalize well across varying motion patterns and sensor noise.

In contrast, deep-learning-based models [1,2,3,4,5,6,7,8,9,10,11,12] have demonstrated a significantly higher performance. For instance, the AM-DLFC and LGBM model [1] and the vRPE + CFEB model (SL 04) both achieved around 96.8% accuracy. The DenseNet121 + MWT model [2] performed even better, with 97.48% accuracy and a high F-score, while the Deep-HAR model [12] nearly saturated the dataset, with 99.98% accuracy. However, these models come with high computational demands, long training times, and the need for GPU support, limiting their use in edge-based or real-time applications.

Our proposed models—GJO-XGB and WARSO-XGB—have achieved 93.55% and 94.02% accuracy, respectively. They have also maintained high F-scores of 92.24 % and 92.88 %, along with corresponding precision values of 92.88% and 93.47% and recall values of 91.74% and 92.40%. Both models have exhibited strong generalization, with a consistently high AUC and ROC performance. However, WARSO-XGB has shown a slight superiority in its average classification performance on unseen data. These models are based on traditional gradient boosting techniques, and the incorporation of metaheuristic optimization has significantly enhanced their performance. This approach has enabled effective feature selection and hyperparameter tuning, resulting in improved generalization and robust classification boundaries. Consequently, our models have outperformed all previous traditional classifiers and have shown competitive results against those of deep learning models while preserving low computational complexity. This makes them highly suitable for deployment in real-world, resource-constrained environments such as mobile or embedded systems. Additionally, our study has identified several important features for HAR using SHAP plots, including range_gyro_x, max_acc_z, mean_gyro_x, range_acc_x, std_acc_z, range_acc_z, max_gyro_y, max_acc_x, and mad_acc_y. These features have shown strong discriminative power in distinguishing between various physical activities.

After conducting external iterations of 10-fold cross-validation using different random seeds, we have observed that the GJO-XGB model achieved its highest test accuracy of 93.80% at a seed of 20, while the WARSO-XGB model yielded a mean accuracy of 94.14% across all seeds. The performance deviations across the different random seeds have remained minimal, as illustrated in Figure 7. Notably, these results are closely aligned with those obtained using a fixed seed of 42, as reported throughout this study. This consistency in the performance, despite variations in data partitioning, has demonstrated the robustness and generalizability of the proposed models. Nevertheless, the Wilcoxon signed-rank test has confirmed that both GJO-XGB (*p* value = 0.005859) and WARSO-XGB (*p* value = 0.001953) have achieved statistically significant improvements in their test accuracy over non-optimized classifiers, as the *p* values are less than 0.05 for each method. Notably, these gains have been obtained while reducing the feature set to 23 features for GJO-XGB and 44 features for WARSO-XGB, highlighting both performance and efficiency benefits. The WARSO-XGB model (random seed: 20) has achieved the best mean training and testing accuracies of 100% and 94.19%, respectively. This has resulted in a performance gap of 5.81% in accuracy between the training and testing phases, indicating an overfitting issue. We have identified that integrating a more diverse dataset has the potential to address this issue, which we plan to resolve in our future work.

We conducted a detailed analysis of the individual predictions to gain deeper insights into the model behavior. Special attention has been given to examining the causes of misclassifications, which has helped identify key features influencing incorrect predictions. This thorough evaluation supports improvement of the models’ robustness and interoperability. To strengthen the discussion of this research, the following questions can be answered:

1. Question: How does statistical feature engineering of time-series sensor data affect model efficiency and generalization in human activity recognition?
  Answer: Statistical feature engineering extracts key characteristics from raw sensor data—such as the mean, median, RMS, SD, and MAD (see Table 4)—which reduces the data dimensionality and computational cost. This simplification helps the model train faster and generalize better, improving the efficiency and performance of human activity recognition.
2. Question: How effectively do metaheuristic algorithms enhance the feature selection for human activity recognition using the KU-HAR dataset?
  Answer: Metaheuristic algorithms (GJO and WARSO) effectively reduced the feature dimensionality while simultaneously tuning the XGBoost hyperparameters, resulting in a more optimized and accurate HAR model.
3. Question: Can an optimized XGB classifier deliver a competitive performance in human activity recognition?
  Answer: Yes, the optimized XGB models (GJO-XGB and WARSO-XGB) achieved a competitive performance, with WARSO-XGB reaching 94.02% accuracy and the optimized metaheuristic algorithms outperforming several traditional models and approaching deep learning benchmarks. Additionally, GJO-XGB achieved 93.55% accuracy using only 23 features, reduced by the MHA. Meanwhile, the performance was increased up to 93.80% (GJO-XGB) and 94.19% (WARSO-XGB) by changing the random seed to 20.
4. Question: To what extent can SHAP interpret model predictions and analyze misclassifications in human activity recognition?
  Answer: SHAP effectively interprets model predictions by identifying influential features through bar plots and explaining the decisions using decision and waterfall plots. It also provides insights into both correct and incorrect classifications.

Future scope: This study focused on the KU-HAR dataset, where MHAs, XGB, and SHAP were utilized for feature optimization, robust activity classification, and interpretability. Future work could explore hybrid architectures that combine deep learning with MHAs to improve the accuracy and generalizability further. Additionally, deploying the models in real-time environments—such as healthcare monitoring systems, elderly fall detection, smart fitness tracking, and occupational safety applications—could validate their effectiveness in real-world settings. Expanding the framework to incorporate multimodal sensor data and testing on larger, cross-domain datasets will enhance its adaptability. Moreover, integrating advanced explainable AI (XAI) techniques beyond SHAP could offer deeper insights into model decisions, which is critical for applications requiring high transparency and trust. As part of our future work, we plan to extend this research by employing additional optimization algorithms to compare the model performance on the current dataset. Furthermore, we aim to validate the generalizability of our proposed approach by applying it to other relevant datasets.

Limitations: In this study, we extracted eight primary statistical features—mean, median, RMS, minimum, maximum, SD, range, and MAD. However, additional mathematical formulations could be adopted to generate more diverse and informative features. The exclusion of such features may limit the richness of the feature space and represent a potential limitation of this research work. The WARSO-XGB model (random seed: 20) has achieved mean training and testing accuracies of 100% and 94.19%, respectively, resulting in a 5.81% gap that indicates overfitting. We have considered integrating a more diverse dataset to address this issue in our future work.

## 5. Conclusions

Human activity recognition has emerged as a technique that leverages machine learning and sensor data to identify human activities. In this study, two novel classification frameworks—GJO-XGB and WARSO-XGB—have been proposed by integrating the traditional Extreme Gradient Boosting algorithm with metaheuristic algorithms: Golden Jackal Optimization and War Strategy Optimization. Informative features have been extracted using simple and widely accepted mathematical expressions from accelerometer and gyroscope sensor data, resulting in a total of 48 engineered features. The WARSO-XGB framework achieved an outstanding performance, with a mean accuracy, F-score, precision, and recall of 94.02%, 92.88%, 93.47%, and 92.40%, respectively, using only 44 selected features. In comparison, GJO-XGB attained a competitive mean accuracy of 93.55% with just 23 features. With external random seeds in 10-fold cross-validation, GJO-XGB and WARSO-XGB recorded accuracies of 93.80% and 94.19%, respectively, at random seed = 20. A SHAP analysis was employed to interpret the model’s predictions, revealing the effectiveness of individual features through the mean SHAP values and feature-wise contributions. Features such as range_gyro_x, mad_acc_x, and mean_gyro_x have shown strong associations with misclassifications, offering valuable insights for enhancing HAR model performance. A limitation of this study is that more complex mathematical features may exist but were not explored. Additionally, we used only the KU-HAR dataset and plan to validate our approach on external datasets in future work.

## Figures and Tables

**Table 1:** A summary of the previously used KU-HAR dataset and its performance.

Type	SL	Author	Model	Accuracy (%)
Traditional Classifier	01	Sikder et al. [9]	RF	89.67
	02	Al-Qaness et al. [10]	AO + RF	88.53
Deep Learning Model	03	Akter et al. [1]	AM-DLFC & LGBM	96.86
	04	Guo et al. [11]	vRPE + CFEB	96.80
	05	Pavliuk et al. [2]	DenseNet121 + MWT	97.48
	06	Kumar et al. [12]	Deep-HAR model	99.98

**Table 2:** Column-wise description of the original dataset structure.

Columns	Description	Columns	Description
1–300	Accelerometer X-axis readings	901–1200	Gyroscope X-axis readings
301–600	Accelerometer Y-axis readings	1201–1500	Gyroscope Y-axis readings
601–900	Accelerometer Z-axis readings	1501–1800	Gyroscope Z-axis readings
1801	Activity class ID (0–17)		
1802	Channel length		
1803	Subsample serial number		

**Table 3:** Activities in the KU-HAR dataset.

Class	ID	Description	Duration	Samples	Subsamples
Stand	0	Standing still	1 min	91	1886
Sit	1	Sitting still	1 min	90	1874
Talk–sit	2	Talking while sitting	1 min	86	1797
Talk–stand	3	Talking while standing	1 min	88	1866
Stand–sit *	4	Sit-to-stand transitions	5 times	339	2178
Lay	5	Lying down	1 min	87	1813
Lay–stand *	6	Lie-to-stand transitions	5 times	148	1762
Pick	7	Picking up object	10 times	105	1333
Jump	8	Jumping in place	10 times	130	666
Push-up	9	Push-ups (wide hands)	5 times	111	480
Sit-up	10	Sit-ups (straight legs)	5 times	121	1005
Walk	11	Walking 20 m	≈12 s	188	882
Walk-back	12	Walking backward	≈20 s	50	317
Walk-circle	13	Walking in circle	≈20 s	35	259
Run	14	Running 20 m	≈7 s	146	595
Stair-up	15	Going upstairs	≈1 min	53	798
Stair-down	16	Going downstairs	≈50 s	57	781
Table tennis	17	Playing table tennis	1 min	20	458

* Postural transition activities.

**Table 4:** Mathematical formulas used for feature extraction.

SL	Feature	Formula
01	Mean	$\mu =\frac{1}{n}\sum_{i=1}^{n}x_{i}$
02	Median	Middle value of sorted $\left\{x_{1},x_{2},\ldots ,x_{n}\right\}$
03	Root Mean Square (RMS)	$\sqrt{\frac{1}{n}\sum_{i=1}^{n}x_{i}^{2}}$
04	Minimum	$\min\{x_{1},x_{2},\ldots ,x_{n}\}$
05	Maximum	$\max\{x_{1},x_{2},\ldots ,x_{n}\}$
06	Standard Deviation (SD)	$\sqrt{\frac{1}{n}\sum_{i=1}^{n}{\left(x_{i}-\mu \right)}^{2}}$
07	Range	$\max\left\{x_{i}\right\}-\min\left\{x_{i}\right\}$
08	Mean Absolute Deviation (MAD)	$\frac{1}{n}\sum_{i=1}^{n}\left|x_{i}-\mu \right|$

**Table 5:** List of 48 features extracted from sensor data.

Serial	Name	Serial	Name	Serial	Name	Serial	Name
0	mean_acc_x	12	max_acc_y	24	mean_gyro_x	36	max_gyro_y
1	median_acc_x	13	std_acc_y	25	median_gyro_x	37	std_gyro_y
2	rms_acc_x	14	range_acc_y	26	rms_gyro_x	38	range_gyro_y
3	min_acc_x	15	mad_acc_y	27	min_gyro_x	39	mad_gyro_y
4	max_acc_x	16	mean_acc_z	28	max_gyro_x	40	mean_gyro_z
5	std_acc_x	17	median_acc_z	29	std_gyro_x	41	median_gyro_z
6	range_acc_x	18	rms_acc_z	30	range_gyro_x	42	rms_gyro_z
7	mad_acc_x	19	min_acc_z	31	mad_gyro_x	43	min_gyro_z
8	mean_acc_y	20	max_acc_z	32	mean_gyro_y	44	max_gyro_z
9	median_acc_y	21	std_acc_z	33	median_gyro_y	45	std_gyro_z
10	rms_acc_y	22	range_acc_z	34	rms_gyro_y	46	range_gyro_z
11	min_acc_y	23	mad_acc_z	35	min_gyro_y	47	mad_gyro_z
48	Target Class: activity						

acc = accelerometer; gyro = gyroscope.

**Table 6:** Prior evaluation of classifiers.

Model	Train F-Score (%)	Test F-Score (%)
CB	98.50	91.90
XGB	100.00	92.55
LGBM	100.00	92.37
GB	99.11	90.33

**Table 7:** Boundary conditions for the frameworks.

Hyperparameter	Name	Type	Range
Number of Estimators	n_estimators ($P_{1}$)	Integer	100 to 300
Learning Rate	learning_rate ($P_{2}$)	Float	0.001 to 0.2
Max Depth	max_depth ($P_{3}$)	Integer	3 to 7
Minimum Child Weight	min_child_weight ($P_{4}$)	Integer	1 to 10
Feature Range	features	Binary Vector (length = 48)	Binary (0 or 1)

**Table 8:** Fold-wise optimized hyperparameters and selected features.

Optimizer	Fold	Features	$P_{1}$	$P_{2}$	$P_{3}$	$P_{4}$
GJO	1	5, 6, 7, 11, 14, 23, 27, 32, 34, 38, 41	279	0.116176736	7	1
	2	5, 10, 15, 20, 32, 34, 39, 41	299	0.110572987	7	1
	3	6, 8, 10, 20, 21, 27, 30, 32, 36	272	0.084533705	7	1
	4	6, 7, 10, 14, 20, 30, 32, 34, 45, 47	186	0.126345040	7	1
	5	5, 7, 10, 14, 15, 34, 45	253	0.193675087	7	1
	6	5, 7, 8, 10, 23, 24, 34, 41	274	0.123926983	7	1
	7	1, 8, 14, 20, 21, 23, 34	280	0.179786016	7	1
	8	5, 10, 14, 24, 34, 45	266	0.132827173	7	1
	9	5, 6, 14, 20, 23, 32, 34, 36, 41, 45	280	0.099581423	7	1
	10	5, 14, 20, 27, 32, 34, 38, 41	278	0.143630097	7	1
WARSO	1	1, 5, 16, 17, 18, 21, 22, 23, 24, 25, 29, 30, 35, 36, 40, 41, 44, 46	225	0.171671644	6	8
	2	1, 5, 15, 16, 17, 21, 23, 25, 26, 29, 30, 32, 35, 36, 37, 40, 41, 44, 46	219	0.177963083	5	2
	3	13, 16, 21, 23, 24, 25, 29, 30, 32, 35, 37, 41, 43, 46	233	0.200000000	6	8
	4	1, 5, 6, 15, 16, 17, 18, 23, 24, 25, 26, 27, 28, 29, 30, 35, 36, 40, 41, 43, 44, 46	214	0.200000000	6	7
	5	5, 15, 16, 17, 18, 21, 23, 24, 25, 26, 29, 30, 35, 36, 40, 41, 43, 44, 46	254	0.177088074	6	9
	6	1, 5, 15, 16, 17, 18, 21, 23, 24, 25, 29, 30, 35, 36, 40, 41, 43, 44, 46	230	0.200000000	6	8
	7	1, 4, 5, 6, 7, 8, 9, 10, 11, 15, 16, 17, 26, 30, 31, 33, 38, 39, 40, 42, 43, 44, 47	208	0.200000000	4	2
	8	2, 5, 9, 10, 11, 13, 14, 16, 31, 32, 33, 34, 36, 38, 40, 44, 46, 47	211	0.172988329	6	2
	9	2, 5, 8, 11, 15, 18, 21, 22, 23, 24, 25, 26, 29, 30, 31, 38, 40, 41, 43	230	0.130140028	5	2
	10	1, 9, 11, 12, 13, 15, 16, 21, 24, 25, 26, 32, 35, 36, 37, 38, 40, 41, 43, 44, 45	211	0.200000000	6	7

**Table 9:** Finalized hyperparameters and selected features.

Optimizer	Features	No. of Features	$P_{1}$	$P_{2}$	$P_{3}$	$P_{4}$
GJO	1, 5, 6, 7, 8, 10, 11, 14, 15, 20, 21, 23, 24, 27, 30, 32, 34, 36, 38, 39, 41, 45, 47	23	280	0.116176736	7	1
WARSO	1, 2, 4, 5, 6, 7, 8, 9, 10, 11, 12, 13, 14, 15, 16, 17, 18, 21, 22, 23, 24, 25, 26, 27, 28, 29, 30, 31, 32, 33, 34, 35, 36, 37, 38, 39, 40, 41, 42, 43, 44, 45, 46, 47	44	230	0.2	6	2

**Table 10:** Model results using fold validation.

	Train					Test			
**Models**	**Fold**	**Accuracy**	**F-Score**	**Precision**	**Recall**	**Accuracy**	**F-Score**	**Precision**	**Recall**
GJO-XGB	1	100%	100%	100%	100%	93.54%	92.28%	93.18%	91.58%
	2	100%	100%	100%	100%	93.41%	92.02%	92.72%	91.44%
	3	100%	100%	100%	100%	93.93%	92.82%	93.42%	92.32%
	4	100%	100%	100%	100%	93.35%	91.93%	92.37%	91.60%
	5	100%	100%	100%	100%	93.67%	92.39%	92.92%	91.98%
	6	100%	100%	100%	100%	93.77%	92.34%	92.52%	92.19%
	7	100%	100%	100%	100%	93.67%	91.85%	92.28%	91.48%
	8	100%	100%	100%	100%	93.35%	92.16%	93.06%	91.46%
	9	100%	100%	100%	100%	93.43%	92.16%	93.20%	91.40%
	10	100%	100%	100%	100%	93.38%	92.46%	93.13%	91.91%
WARSO-XGB	1	100%	100%	100%	100%	94.17%	93.03%	93.84%	92.39%
	2	100%	100%	100%	100%	94.47%	93.54%	94.04%	93.12%
	3	100%	100%	100%	100%	94.23%	93.06%	93.57%	92.62%
	4	100%	100%	100%	100%	93.72%	92.47%	92.72%	92.32%
	5	100%	100%	100%	100%	93.98%	92.75%	93.25%	92.35%
	6	100%	100%	100%	100%	93.65%	92.28%	92.79%	91.85%
	7	100%	100%	100%	100%	93.83%	92.15%	92.69%	91.71%
	8	100%	100%	100%	100%	93.88%	92.97%	93.46%	92.55%
	9	100%	100%	100%	100%	94.23%	93.24%	94.22%	92.52%
	10	100%	100%	100%	100%	94.07%	93.28%	94.11%	92.61%

**Table 11:** Standard deviation of performance metrics across 10-fold validation.

Model	Train				Test			
	**Accuracy**	**F-Score**	**Precision**	**Recall**	**Accuracy**	**F-Score**	**Precision**	**Recall**
GJO-XGB	0.000	0.000	0.000	0.000	0.200	0.285	0.388	0.336
WARSO-XGB	0.000	0.000	0.000	0.000	0.259	0.455	0.589	0.399

**Table 12:** Mean results of models using 10-fold validation.

	Train				Test			
**Models**	**Accuracy**	**F-Score**	**Precision**	**Recall**	**Accuracy**	**F-Score**	**Precision**	**Recall**
GJO-XGB	100%	100%	100%	100%	93.55%	92.24%	92.88%	91.74%
WARSO-XGB	100%	100%	100%	100%	94.02%	92.88%	93.47%	92.40%

**Table 13:** The AUC for each fold and the mean across 10-fold cross-validation.

Model	Fold 1	Fold 2	Fold 3	Fold 4	Fold 5	Fold 6	Fold 7	Fold 8	Fold 9	Fold 10	Mean
GJO-XGB	0.99675	0.99715	0.99754	0.99729	0.99663	0.99736	0.99816	0.99724	0.99703	0.99579	0.99709
WARSO-XGB	0.99712	0.99717	0.99736	0.99710	0.99718	0.99754	0.99805	0.99651	0.99639	0.99547	0.99699

**Table 14:** Model results using fold validation across different random seeds (R. Seed = random seed).

		Train (%)				Test (%)			
**Models**	**R. Seed**	**Accuracy**	**F-Score**	**Precision**	**Recall**	**Accuracy**	**F-Score**	**Precision**	**Recall**
GJO-XGB	5	100	100	100	100	93.57	92.25	92.94	91.71
	10	100	100	100	100	93.58	92.28	92.85	91.81
	15	100	100	100	100	93.60	92.35	92.96	91.86
	20	100	100	100	100	93.80	92.54	93.19	92.02
	25	100	100	100	100	93.64	92.31	92.97	91.81
	30	100	100	100	100	93.60	92.32	92.86	91.91
	35	100	100	100	100	93.64	92.37	92.99	91.92
	40	100	100	100	100	93.59	92.22	92.90	91.67
	45	100	100	100	100	93.56	92.28	92.92	91.76
	50	100	100	100	100	93.64	92.34	92.95	91.85
WARSO-XGB	5	100	100	100	100	94.00	92.80	93.36	92.37
	10	100	100	100	100	93.93	92.72	93.21	92.32
	15	100	100	100	100	94.05	92.83	93.35	92.40
	20	100	100	100	100	94.19	93.11	93.64	92.69
	25	100	100	100	100	93.94	92.69	93.24	92.26
	30	100	100	100	100	93.99	92.74	93.26	92.34
	35	100	100	100	100	94.02	92.82	93.38	92.40
	40	100	100	100	100	93.92	92.64	93.15	92.21
	45	100	100	100	100	93.95	92.74	93.30	92.29
	50	100	100	100	100	94.12	93.00	93.60	92.51

**Table 15:** Training and testing times (in seconds) for each fold.

	GJO-XGB Framework		WARSO-XGB Framework	
**Fold**	**TrT (s)**	**TsT (s)**	**TrT (s)**	**TsT (s)**
1	31.88	0.62	42.59	0.54
2	28.31	0.62	29.01	0.62
3	41.44	0.81	29.06	0.43
4	41.59	0.71	30.77	0.42
5	41.85	1.08	29.22	0.47
6	45.59	0.79	29.67	0.99
7	40.84	0.85	28.54	0.39
8	40.99	0.72	30.06	0.43
9	41.03	0.82	30.25	0.41
10	40.50	1.08	29.19	0.43
Mean	39.40	0.81	30.84	0.51

**Table 16:** A comparison table of previous studies and our findings.

SL	Model	Accuracy (%)	F-Score (%)	Precision (%)	Recall (%)
Traditional Classifier Models					
01 [9]	RF	89.67	87.59	–	–
02 [10]	AO + RF	88.53	–	–	–
Deep Learning Models					
03 [1]	AM-DLFC and LGBM	96.86	96.92	–	–
04 [11]	vRPE + CFEB	96.80	97.50	–	–
05 [2]	DenseNet121 + MWT	97.48	97.52	97.62	97.41
06 [12]	Deep-HAR model	99.98	98.96	97.38	100
Proposed Models					
01	Only XGB	93.81	92.64	93.23	92.16
01	GJO-XGB	93.55	92.24	92.88	91.74
02	WARSO-XGB	94.02	92.88	93.47	92.40
03	GJO-XGB (random seed = 20)	93.80	92.54	93.19	92.02
04	WARSO-XGB (random seed = 20)	94.19	93.11	93.64	92.69

## Data Availability

The dataset is available in the Mendeley Data Repository (dataset name: KU-HAR: An Open Dataset for Human Activity Recognition): https://doi.org/10.17632/45f952y38r.5, https://data.mendeley.com/datasets/45f952y38r/5. Accessed on 4 July 2025.
